# Isolation, identification, and complete genome sequence of a bovine adenovirus type 3 from cattle in China

**DOI:** 10.1186/1743-422X-8-557

**Published:** 2011-12-22

**Authors:** Yuan-Mao Zhu, Zuo Yu, Hong Cai, Yu-Ran Gao, Xiu-Mei Dong, Zhao-Li Li, Hong-Fei Shi, Qing-Feng Meng, Chuang Lu, Fei Xue

**Affiliations:** 1 Division of Livestock Infectious Diseases, State Key Laboratory of Veterinary Biotechnology, Harbin Veterinary Research Institute of Chinese Academy of Agricultural Sciences, No. 427 Maduan Street, Nan Gang District, Harbin 150001, Heilongjiang Province, PR China; 2 Department of Ophthalmology, First Affiliated Hospital of Harbin Medical University, Heilongjiang, Harbin, China

**Keywords:** Bovine adenovirus type 3, Cattle, Complete genome, DNA binding protein

## Abstract

**Background:**

Bovine adenovirus type 3 (BAV-3) belongs to the *Mastadenovirus *genus of the family *Adenoviridae *and is involved in respiratory and enteric infections of calves. The isolation of BAV-3 has not been reported prior to this study in China. In 2009, there were many cases in cattle showing similar clinical signs to BAV-3 infection and a virus strain, showing cytopathic effect in Madin-Darby bovine kidney cells, was isolated from a bovine nasal swab collected from feedlot cattle in Heilongjiang Province, China. The isolate was confirmed as a bovine adenovirus type 3 by PCR and immunofluorescence assay, and named as HLJ0955. So far only the complete genome sequence of prototype of BAV-3 WBR-1 strain has been reported. In order to further characterize the Chinese isolate HLJ0955, the complete genome sequence of HLJ0955 was determined.

**Results:**

The size of the genome of the Chinese isolate HLJ0955 is 34,132 nucleotides in length with a G+C content of 53.6%. The coding sequences for gene regions of HLJ0955 isolate were similar to the prototype of BAV-3 WBR-1 strain, with 80.0-98.6% nucleotide and 87.5-98.8% amino acid identities. The genome of HLJ0955 strain contains 16 regions and four deletions in inverted terminal repeats, E1B region and E4 region, respectively. The complete genome and DNA binding protein gene based phylogenetic analysis with other adenoviruses were performed and the results showed that HLJ0955 isolate belonged to BAV-3 and clustered within the *Mastadenovirus *genus of the family *Adenoviridae*.

**Conclusions:**

This is the first study to report the isolation and molecular characterization of BAV-3 from cattle in China. The phylogenetic analysis performed in this study supported the use of the DNA binding protein gene of adenovirus as an appropriate subgenomic target for the classification of different genuses of the family *Adenoviridae *on the molecular basis. Meanwhile, a large-scale pathogen and serological epidemiological investigations for BVA-3 infection might be carried out in cattle in China. This report will be a good beginning for further studies on BAV-3 in China.

## Introduction

Bovine adenovirus type 3 (BAV-3) belongs to the *Mastadenovirus *genus of the family *Adenoviridae *and is involved in respiratory and enteric infections of calves [1]. Bovine adenoviruses (BAVs) cause a variety of clinical signs including conjunctivitis, pneumonia, diarrhea, and polyarthritis [2,3]. BAVs are classified into ten serotypes [4]. The serotypes of BAV-1, -2, -3, -9 and -10 belong to *Mastadenovirus *genus, and the serotypes of BAV-4, -5, -6, -7, and -8 belong to *Atadenovirus *genus http://www.ictvdb.org. These ten serotypes are also divided into two groups on the basis of the differences in their biological and serological distinctiveness [4,5]. The members of subgroup 1 bovine adenoviruses (BAV-1, -2, -3, and -9) grow well in established bovine cell lines and contain common complement-fixing antigens, which cross-react with the members of other mastadenoviruses in the complement fixation tests. However, the members of subgroup 2 (BAV-4, -5, -6, -7, -8, and -10) do not cross-react with any other mammalian adenovirus in the complement fixation test and can be propagated exclusively in low-passage cultures of calf testicular or thyroid cells [6,7].

BAV-3, a member of subgroup 1, is considered one of the important respiratory tract pathogens of cattle, particularly newborn calves [8]. Clinical signs include pyrexia, respiratory distress, and nasal and conjunctival discharges. BAV-3 was firstly isolated by Darbyshire and coworkers in Britain [9]. Like other adenoviruses, BAV-3 is a nonenveloped icosahedral particle of 75-80 nm in diameter and has a double-stranded linear genomic DNA [10]. The E1, E3, and E4 regions and its complete genome sequence of BAV-3 have been described [6,11,12]. Serologic surveys indicated widespread distribution of BAV throughout the world. The detection, isolation or serological evidence of BAV-3 has not been reported in China. However, there were many cases in cattle showing similar clinical signs to BAV-3 infection in China in 2009. Then we made an attempt to isolate the virus with nasal swabs from cattle in Heilongjiang Province, China, and isolated a virus strain using Madin-Darby bovine kidney (MDBK) cell cultures from bovine nasal swabs. The virus isolate was further characterized for some biological properties, partial and the complete genome sequencing for the isolate and phylogenetic analysis.

## Results

### BAV-3 isolation and confirmation

Nasal swabs collected from a group of feedlot cattle with acute respiratory disease were inoculated into cultures of MDBK cells, and the third passage of one specimen caused obvious cytopathic effect (CPE) in MDBK cells. Compared with normal control MDBK cells, the MDBK cells inoculated with the specimen rounded up and cytoplasmic bridges formed after an incubation period of five days (data not shown). The CPE caused by the specimen was similar to that caused by a BAV-3 isolate FSO-213 [13]. The virus isolate was designated as HLJ0955.

Viral genomic DNA was extracted from the culture supernatant inoculated with isolate HLJ0955 and amplified by polymerase chain reaction (PCR) with the specific primers E2Afwd and E2Aseq1 for BAV-3. Fragment consistent with the expected size of 644 bp (base pair) was obtained from the amplification of isolate HLJ0955 (data not shown), which caused typical CPE in MDBK cells. The amplified product was purified, cloned and sequenced. Blast search revealed that the sequence of the amplified fragment was related to BAV-3 with 95% nucleotide identity.

The immunofluorescence staining of the MDBK cells inoculated with isolate HLJ0955 and control MDBK cells with BAV-3 specific polyclonal antibody was also done. The immunofluorescence was detected in the cytoplasm of MDBK cells after inoculation with isolate HLJ0955, but there was no immunofluorescence in control MDBK cells (data not shown). All of these results demonstrated that the isolate HLJ0955 was BAV-3.

### Morphological determination of strain HLJ0955 using electric microscopy

**Figure 1:**
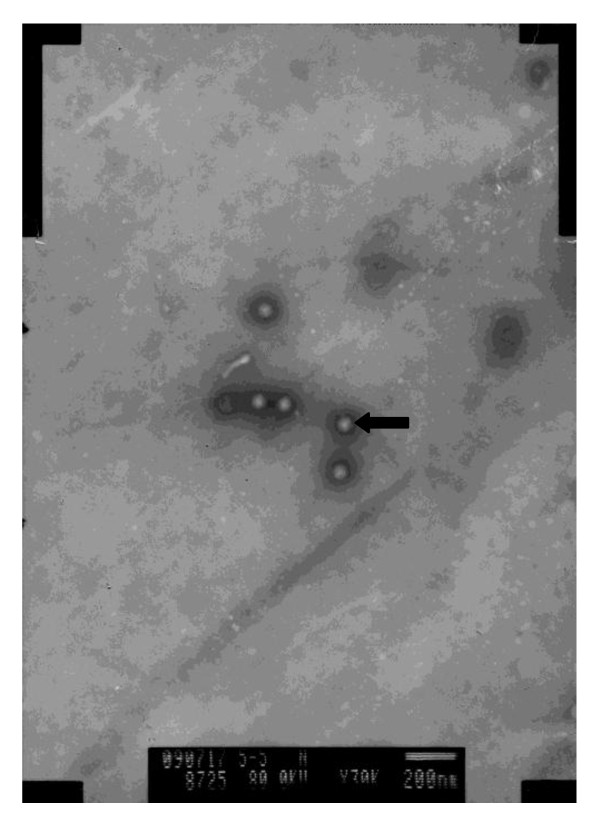
**Electron micrograph of the newly isolated BAV-3 strain HLJ0955 in MDBK cell cultures displaying typical adenovirus morphology**. Typical virions of approximately 75 nm in diameter were observed (×30, 000).

Typical virions of approximately 75 nm in diameter were observed in negative-stain preparations of MDBK cells inoculated with the isolate HLJ0955 (Figure 1).

### Full-length sequencing of newly isolated HLJ0955 strain

The complete genome sequence for HLJ0955 was determined. The entire DBP gene sequence in E2A region and the complete genome sequence of HLJ0955 analyzed in this work were deposited in GenBank under following accession number: JN381195. The complete genome of isolate HLJ0955 is 34,132 nucleotides (nt) in length, which is 314 bases shorter than the previously identified BAV-3 strain WBR-1 genome (34,446 nt). The sequence of the Chinese BAV-3 isolate HLJ0955 has a G+C content of 53.6%, which is similar to the G+C content of the prototype of BAV-3 WBR-1 strain (54.0%). The region, name, coding sequence (CDS) and length of genes of Chinese BAV-3 HLJ0955 strain were summarized in Table 1. To further characterize the molecular structure of the HLJ0955 genome and to determine how it is related to the prototype of BAV-3 WBR-1 strain, we compared the nucleotide and putative amino acid sequences of BAV-3 HLJ0955 strain with those of BAV-3 WBR-1 strain retrieved from GenBank (Table 1). The full-length nucleotide homology between HLJ0955 and WBR-1 strains was 70.2%. The CDS for gene regions of HLJ0955 isolate were similar to the prototype of BAV-3 WBR-1 strain, with 80.0-98.6% nucleotide and 87.5-98.8% amino acid identities (Table 1).

**Table 1:** Summary of the region, name, CDS and length of genes of Chinese BAV-3 HLJ0955 strain and the nucleotide and putative amino acid identities between HLJ0955 and the prototype of BAV-3 WBR-1 strain

Region	Gene	CDS (HLJ0955)	Gene length (bp)	Nucleotide identity with WBR-1 (%)	Amino acid identity with WBR-1 (%)
ITR	ITR	1-85	85	ND^b^	ND
		34048-34132	85	ND	ND

E1A	211R	560-1169, 1277-1302(join)	636	98.6	98.6

E1B	157R	1395-1868	474	98.0	98.1
	420R	1769-3031	1263	96.4	97.9

pIX	pIX	3117-3494	378	94.8	97.6

IVa2	IVa2	3531-4661(complement^a^)	1131	96.3	98.1

E2B	Pol	4640-7711(complement)	3072	95.9	96.6
	TP	7930-9882(complement)	1953	83.9	94.8

L1	52K	9912-10907	996	96.3	97.9
	IIIA	11022-12737	1716	93.0	96.8
	III	12852-14300	1449	97.5	96.5
	pVII	14363-14878	516	97.3	97.7

L2	pV	14995-16266	1272	94.1	87.6

L3	pX	16393-16632	240	83.6	98.8

L4	pVI	16792-17586	795	97.8	87.5

L5	Hexon	17733-20459	2727	80.0	94.1
	Protease	20488-21102	615	95.7	98.0

E2A	DBP	21219-22517(complement)	1299	96.1	98.4

L6	100K	22544-25096	2553	96.5	96.0
	33K	24729-25553	825	96.9	92.7
	pVIII	25732-26382	651	97.2	97.2

E3	284R	26441-27295	855	95.3	90.9
	121R	27306-27671	366	97.0	97.5

L7	Fiber	27898-30828	2931	93.4	92.7

E4	ORF5	30906-31565(complement)	660	97.4	97.3
	ORF4	31504-31935(complement)	432	96.0	93.1
	ORF3	31957-32763(complement)	807	96.3	97.0
	ORF2	33028-33237(complement)	210	95.7	95.7
	ORF1	33250-33675(complement)	426	91.7	90.8

^a ^represents complimentary strand; ^b ^represents "not done"

### Adenovirus subtyping using DBP gene

**Figure 2:**
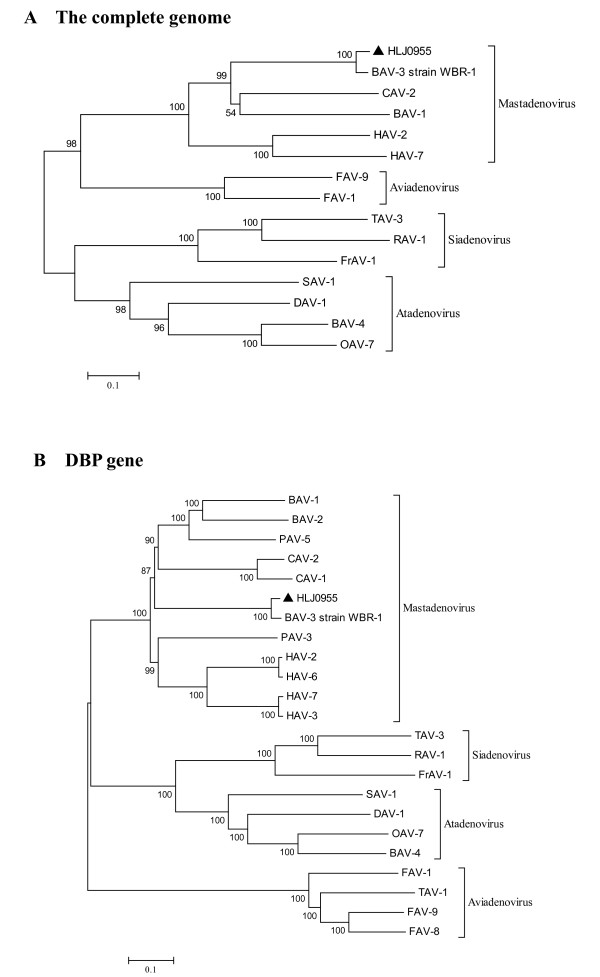
**Phylogenetic analysis of the complete genome was created using the complete genome sequence of the HLJ0955 isolate in this work, and 14 BAVs and adenovirus reference strains from other animals or human beings retrieved from GenBank (A, the complete genome)**. Phylogenetic analysis of the DBP gene was created using the nucleotide sequences of the Chinese BAV-3 isolate HLJ0955 in this work, and 22 BAVs and adenovirus reference strains from other animals or human beings retrieved from GenBank (B, DBP gene). The phylogenetic tree was prepared using the Neighbor-Joining method and bootstrap testing. Numbers over branches indicate the percentage of 1000 bootstrap replicates that support each phylogenetic branch. The GenBank accession numbers of adenovirus reference strains are as follows: BAV-1 (NC_006324), BAV-2 (AF252854), BAV-3 strain WBR-1 (AF030154), BAV-4 (AF036092), canine adenovirus-1 (CAV-1) (AC_000003), CAV-2 (U77082), duck adenovirus-1 (DAV-1) (AC_000004), fowl adenovirus-1 (FAV-1) (AC_000014), FAV-8 (AF083975), FAV-9 (AF083975), frog adenovirus-1 (FrAV-1) (NC_002501), human adenovirus-2 (HAV-2) (AC_000007), HAV-3 (DQ086466), HAV-6 (FJ349096), HAV-7 (AY594256), ovine adenovirus-7 (OAV-7) (U40839), porcine adenovirus-3 (PAV-3) (AF083132), PAV-5 (AF289262), raptor adenovirus-1(RAV-1) (NC_015455), snake adenovirus-1 (SAV-1) (DQ106414), turkey adenovirus-1 (TAV-1) (NC_014564), TAV-3 (AC_000016).

A phylogenetic tree constructed with the full-length genomes of different adenoviruses showed that HLJ0955 strain clustered with BAV-3 WBR-1 strain with 100% bootstrap value and also clustered within *Mastadenovirus *genus with some adenoviruses with 100% bootstrap value (Figure 2A). The other adenoviruses contained in phylogenetic tree created from the full-length genome data clustered within four different genuses of *Adenoviridae *family, namely *Mastadenovirus*, *Aviadenovirus*, *Atadenovirus *and *Siadenovirus *genuses, respectively and their branching patterns were in agreement with presently accepted classification of four different genuses of the family *Adenoviridae*. Meanwhile, a phylogenetic analysis was performed with the entire coding sequence of DBP gene in E2A region (Figure 2B). The analysis showed that HLJ0955 strain clustered with BAV-3 WBR-1 strain with 100% bootstrap value and also clustered within *Mastadenovirus *genus with some adenoviruses with 100% bootstrap value. The other adenoviruses contained in phylogenetic tree created from the DBP gene nucleotide data clustered within four different genuses of *Adenoviridae *family and their branching patterns were also in agreement with presently accepted classification of four different genuses of the family *Adenoviridae*. These analysis results showed that the phylogenetic tree created from the DBP gene nucleotide data was in agreement with the phylogenetic tree created from the complete genome sequence.

## Discussion

A virus strain, showing cytopathic effect in MDBK cells, was isolated from a bovine nasal swab collected from feedlot cattle in Heilongjiang Province, China in 2009. The isolate was confirmed as a bovine adenovirus type 3 by PCR and immunofluorescence assay, and named as HLJ0955. As well, typical virions were observed in negative-stain preparations of MDBK cells inoculated with the isolate HLJ0955 (Figure 1). To date, only the complete genome sequence of prototype of BAV-3 WBR-1 strain has been reported. In order to further analyze the Chinese BAV-3 isolate HLJ0955, the complete genome sequence for HLJ0955 was determined. Compared with the prototype of BAV-3 WBR-1 strain, the genome of BAV-3 HLJ0955 strain contains 16 regions and four deletions in inverted terminal repeats (ITR), E1B region and E4 region, respectively. The ITR of isolate HLJ0955 is 85 nt in length, which is 110 bases shorter than the previously identified BAV-3 strain WBR-1 ITR (195 nt). A 46 bp deletion occurs in ITR from the left end of the HLJ0955 genome, which is corresponding to the nt 132 to 182 from the left end of the WBR-1 genome. As well, another 85 bp deletion also occurs in ITR from the left end of the HLJ0955 genome, which is corresponding to the nt 34282 to 34366 from the left end of the WBR-1 genome. Whether or not the deletions in ITR of HLJ0955 affect virus replication need further investigations. Interestingly, a 72 bp internal deletion in the left inverted terminal repeat of the bovine adenovirus type 3 mutant BAV3c29 was also found and did not affect virus replication [14]. A 35 bp deletion occurs in E1B region, which is corresponding to the nt 1370 to 1404 from the left end of the WBR-1 genome. The deletion in E1B region did not affect the coding region of E1B product of HLJ0955. The last 145 bp deletion immediately in E4 open reading frame (ORF) 1 region, which is corresponding to the nt 33471 to 33615 from the left end of the WBR-1 genome. Compared with the prototype of BAV-3 WBR-1 strain, the deleted nucleotides of BAV-3 HLJ0955 strain are totally 311 bp. The deletion in E4 ORF1 region caused minor changes of the ORF1 product of HLJ0955. The E4 region of BAV-3 lies near the right end of the genome (nt 30932c to 33950c) and consists of five ORFs [6]. The proteins encoded by the E4 region are involved at several levels of regulation of cellular and viral gene expression, viral DNA replication, late viral assembly, E2 expression, and adeno-associated virus helper function [15-18]. It has been suggested that individual ORFs of the BAV-3 E4 can be deleted and are nonessential for viral replication [19]. But the deletion of HLJ0955 strain in E4 region did not result in any deletion of five ORF products of HLJ0955 strain.

To better understand the genetic relationships and evolution of HLJ0955 strain with other adenoviruses, phylogenetic analysis were performed with the full-length genomes and entire coding sequence of DBP gene in E2A region of different adenoviruses. The analysis showed that HLJ0955 strain clustered with BAV-3 WBR-1 strain with 100% bootstrap value and also clustered within *Mastadenovirus *genus with some adenoviruses with 100% bootstrap value (Figure 2A and 2B). The other adenoviruses contained in phylogenetic tree clustered within four different genuses of *Adenoviridae *family and their branching patterns were in agreement with presently accepted classification of four different genuses of the family *Adenoviridae*. These results indicated that the phylogenetic tree created from subgenomic data of the DBP gene nucleotide sequences was in agreement with the phylogenetic tree created from the complete genome sequences. As well, phylogenetic reconstruction with DBP gene nucleotide data of human adenovirus also provided meaningful inferences in molecular characterization of human adenoviruses [20]. The determinations of phylogenetic relationships by comparisons of sequence data play a major role in the classification of viruses within a particular genus in addition to serological tests [6]. The phylogenetic analysis results presented in this study supported the use of the DBP gene of adenovirus as an appropriate subgenomic target for the classification of different genuses of the family *Adenoviridae *on the molecular basis. These results may provide insight into the molecular properties of HLJ0955 strain and open a new way to further studies.

The isolation of BAV-3 has not been reported prior to this study in China. An important factor with bovine adenovirus isolation is that a fairly large number of serial blind passages of the material is often necessary before a characteristic CPE begins to develop [13]. We have collected numerous nasal swabs from feedlot cattle with acute respiratory disease and finally succeeded to isolate the HLJ0955 strain in 2009. Bovine respiratory disease complex (BRDC) is a major problem for cattle and it continues to cause serious economic losses for the global cattle industry. The causes of BRDC are multiple and complex, but the three factors of stress, viral infection and bacterial infection are almost always involved in cases of severe disease. From Autumn in 2008 on, it was reported that a few herds of calves showed acute respiratory disease after a long distance transportation for sales in China. The sick calves were treated with antibiotics, but recovered very slowly and some of them died. Then viral agents were suspected for involvement in the calf pneumonia and bovine parainfluenza virus type 3 and bovine viral diarrhea virus were detected [21,22]. However, the viral agents involved in BRDC still need further investigations in China. This is the first report about the detection and isolation of BAV-3 in China. On the basis of this work, a broad pathogen epidemiological investigation of BAV-3 might be carried out and serological methods would be established for detecting antibodies against BAV-3 in China. Vaccine for BAV-3 might be developed using the new isolate HLJ0955 of BAV-3. All of these efforts would greatly improve the investigations on BRDC in China. On the other hand, recombinant BAV-3 is being developed as a live vector for animal vaccination and for human gene therapy [12,23]. Therefore the BAV-3 strain HLJ0955 might be developed as an appropriate expression vector.

## Conclusions

This is the first study to report the isolation and molecular characterization of BAV-3 from cattle in China. The phylogenetic analysis performed in this study supported the use of the DNA binding protein gene of adenovirus as an appropriate subgenomic target for the classification of different genuses of the family *Adenoviridae *on the molecular basis. Meanwhile, a large-scale pathogen and serological epidemiological investigations for BVA-3 infection might be carried out in cattle in China. This report will be a good beginning for further studies on BAV-3 in China.

## Materials and methods

### Preparation of samples and virus isolation

Nasal swabs from feedlot cattle with acute respiratory disease were collected from Heilongjiang Province, China in 2009. The swabs were centrifuged for 5 min at 800 × g and the supernatant fluids were used for virus isolation. MDBK cells were grown in minimum essential medium (MEM, GIBCO) supplemented with 10% heat inactivated fetal bovine serum (BIOCHROM AG, German). The supernatants of nasal swabs (100 μl) were inoculated into each well of MDBK cells cultured in 24-well cell culture plates and incubated for 1 h at 37°C. Then the inoculations were discarded and 500 μl MEM supplemented with 4% heat inactivated fetal bovine serum were added. The cell cultures were frozen and thawed three times and passaged three to five times at seven days interval. Harvest the cell cultures by freezing and thawing them for three times when CPE appeared in monolayer of MDBK cells. Then the isolates were further identified.

### PCR detection and nucleotide sequence analysis

Oligonucleotide primers for BAV-3 detection and identification were designed from the DNA binding protein (DBP) gene sequence in E2A region of BAV-3 strain WBR-1 (GenBank accession number AF030154). The sequence of two pairs of primers, designed E2Afwd and E2Aseq1, was located in E2A region of BAV-3. The primers E2Afwd (5'-GAG ATG GAT GTG AAC AGC GA-3') and E2Aseq1 (5'-ACA TTC TGA TGC TGG TAC TG-3') amplified an approximately 644 bp product from the BAV-3 DNA.

Viral genomic DNA was extracted from 500 μl of infected culture supernatant containing 0.2 mg/ml proteinase K (AMRESCO, USA) and 0.5% sodium dodecyl sulfate, which was incubated at 37°C for two hours. The digested solution was extracted once with phenol and twice with chloroform-isoamyl alcohol (24:1). The extracted DNA was precipitated by the addition of two volumes of absolute ethanol, recovered by centrifugation, and dissolved in 100 μl of TE buffer (10 mM Tris-HCl (pH 7.4) and 1 mM EDTA). The dissolved DNA was used as a template for PCR. The amplification of viral DNA by PCR was carried out in a total volume of 50 μl containing 20 mM Tris-HCl (pH8.4), 50 mM KCl, 3 mM MgCl_2_, 0.5 mM dNTP, 200 pmol of each primer E2Afwd and E2Aseq1, 10 μl extracted DNA and 2.5 U EX Taq DNA polymerase (Takara). The reaction was heated in a thermocycle for 3 min at 93°C and then submitted to 30 cycles of amplification. The conditions of amplification were 45 s at 95°C, 50 s at 55°C, and 1 min at 72°C. The final extension step was done at 72°C for 10 min.

The PCR products were separated by electrophoresis in 1.5% agarose gel in Tris-acetate EDTA buffer and the expected band was excised and recovered from the agarose gel using the Watson gel extraction kit (Watson, China). The purified PCR product was ligated into the pGEM-T vector (Promega) using T/A cloning, and the competent *Escherichia coli *strain DH5α was transformed with the ligation products following the manufacturer's instruction. The positive colonies (three of each sample), screened by blue-white color reaction on X-gal containing plates, were sequenced with the M13+ and M13- sequencing primers. DNA sequencing was performed on an ABI automated A373 sequencer. Sequences were identified using the BLAST search program http://www.ncbi.nlm.nih.gov/blast/Blast.cgi.

### Electron microscope observation

MDBK cells infected with the virus isolate were harvested by freezing and thawing for three times. One ml of the harvested cell cultures was centrifuged for 5 min at 800 × g. The supernatant was transferred into a new eppendorf tube and centrifuged for 10 min at 13,400 × g. Then make negative-stain preparations for transmission electron microscope observation. The observed virions were photographed and analyzed.

### Identification of the isolate by immunofluorescent assay

MDBK cell monolayer grown on 96-well cell culture plates was fixed in 80% chilled acetone for 20 min about 36 h after virus inoculation. After washing out excess unbound reagent with phosphate buffered saline (PBS), the fixed cells were incubated with anti-BAV-3 polyclonal antibody conjugated to FITC (Fluorescein isothiocyanate) (VMRD Inc., USA) at 37°C for 30 min in a humid chamber. After washing with PBS, the cells were examined by fluorescence microscope.

### Sequence analysis of the complete genome for HLJ0955 isolate and phylogenetic analysis

To further characterize the Chinese isolate HLJ0955, the complete genome sequence for isolate HLJ0955 was determined. Forty-two primer sets were designed to amplify overlapping regions of the complete BAV-3 strain HLJ0955 genome. The amplified fragments were harvested and sequenced as described for the E2A region. The complete HLJ0955 genome sequence was compiled from overlapping sequences of the HLJ0955 amplicons. Comparative analysis with the BAV-3 strain WBR-1 genome sequence from GenBank was used to identify coding regions, and putative amino acid sequences were created following BLASTX search routines. Nucleotide and putative amino acid sequence alignments were created using the computer program DNAStar (DNAStar Inc., Madison, WI) with BAV-3 strain WBR-1 sequence retrieved from GenBank (Table 1).

Phylogenetic reconstructions for genetic analysis of the Chinese isolate HLJ0955 were compiled using the complete genome sequence for the isolate HLJ0955 and entire nucleotide coding sequence of DBP gene in E2A region (nucleotide 21284c-22582c of BAV-3 strain WBR-1 genome). Additional sequences from representative isolates of previously identified BAVs and adenoviruses from other animals or human beings were included into phylogenetic analysis. Nucleotide sequences were aligned using the Clustal W program. Phylogenetic and molecular evolutionary analyses were conducted using MEGA version 4.0 [24]. Bootstrap values were calculated on 1000 replicates of the alignment.

## List of Abbreviations

BAV: bovine adenovirus; BAV-3: bovine adenovirus type 3; bp: base pair; BRDC: bovine respiratory disease complex; CDS: coding sequence; CPE: cytopathic effect; DBP: DNA binding protein; FITC: fluorescein isothiocyanate; ITR: inverted terminal repeat; MDBK: Madin-Darby bovine kidney; MEM: minimum essential medium; nt: nucleotide; ORF: open reading frame; PBS: phosphate buffered saline; PCR: polymerase chain reaction.

## Competing interests

The authors declare that they have no competing interests.

## Authors' contributions

YMZ, and ZY carried out the isolation and identification of the Chinese BAV-3 isolate HLJ0955. HC made the negative-stain preparations for HLJ0955. YRG, XMD, and ZLL carried out PCR amplifications, cloning and sequencing for HLJ0955. HFS, and QFM carried out sequence alignment and phylogenetic analysis. CL participated in its design and coordination. FX designed the study and wrote the manuscript. All authors read and approved the final manuscript.
